# Thoracic wall muscle metastasis from pancreatic cancer

**DOI:** 10.1186/s40792-017-0393-0

**Published:** 2018-01-25

**Authors:** Kenji Shimizu, Daisuke Hashimoto, Naoki Umezaki, Shigeki Nakagawa, Kensuke Yamamura, Akira Chikamoto, Fujio Matsumura, Hideo Baba

**Affiliations:** 1 Department of Gastroenterological Surgery, Kumamoto University Graduate School of Medical Sciences, 1-1-1 Honjo, Kumamoto City, 860-8556 Japan; 2 Department of Gastroenterological Surgery, Omuta Tenryo Hospital, 1-100 Tenryo, Omuta City, 836-8566 Japan

**Keywords:** Pancreatic cancer, Muscle metastasis, Operation

## Abstract

Skeletal muscle metastasis from pancreatic cancer is rare. We present a 72-year-old female patient with unresectable pancreatic tail cancer. Fifteen months after the introduction of the chemoradiotherapy, an 18-mm elastic hard tumor was found in her right chest wall and resected after confirmation of no other metastatic lesions. Postoperative pathological examination diagnosed it as a muscle metastasis from the pancreatic cancer, and the patient has since been continuing chemotherapy for 10 months. A review of the literature regarding skeletal muscle metastasis from pancreatic cancer is also presented.

## Background

Pancreatic cancer’s metastasis usually occurs in the liver, lung, peritoneum, or bones [1]. Metastases of malignant tumors to the muscle tissues of the skeletal system are very rare [2–4]. It was reported in literature that muscle tissue metastases accounted for less than 1% [5]. Lung cancer were reported the most frequent primaries which developed skeletal muscle metastasis, followed by gastrointestinal tumors, urological tumors, and genital tumors [6]. We would like to present a rare case of metastatic pancreatic cancer to the chest wall muscle, and a review of the literature regarding skeletal muscle metastasis (SMM) from pancreatic cancer.

## Case presentation

**Fig. 1:**
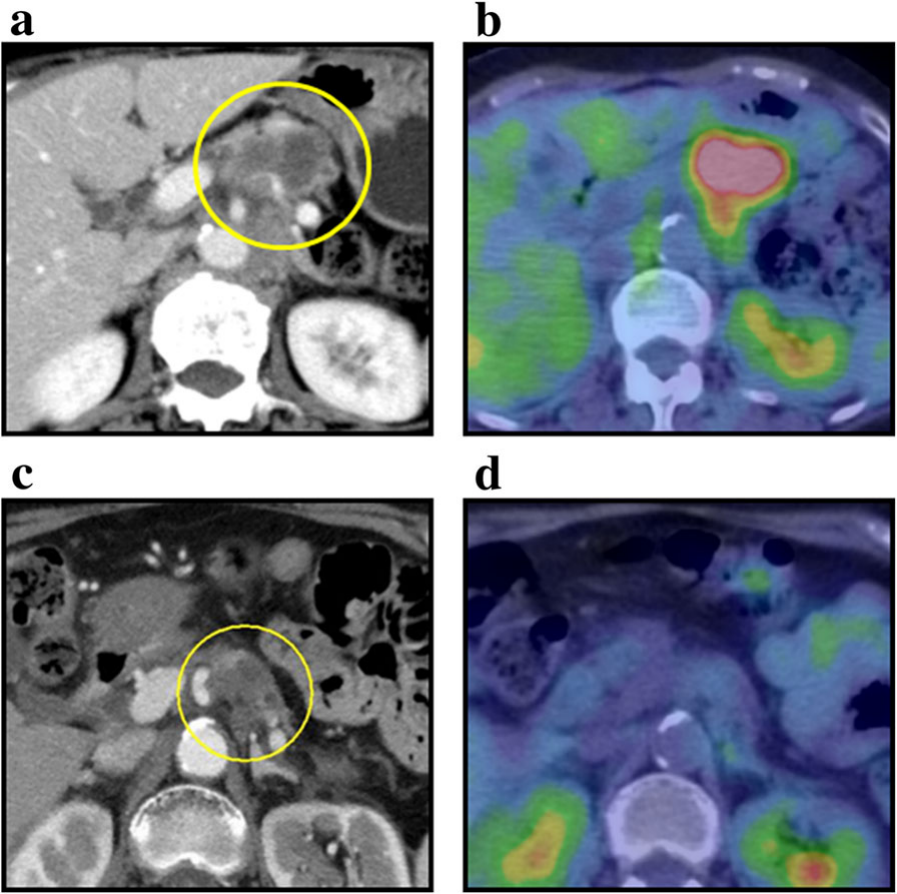
An enhanced CT revealed a 45-mm tumor in the body of pancreas (**a**). The SUV (max) of the tumor was 6.2 in a PET-CT (**b**). After the chemoradiation for 15 months, the size of the primary tumor was reduced to 25 mm (**c**). The abnormal uptake in a PET-CT disappeared (**d**)

A 72-year-old female was investigated because of abdominal pain. An enhanced computed tomography (CT) revealed a 45-mm tumor in the body of the pancreas (Fig. 1a). The maximal standardized uptake value (SUV (max)) of the tumor was 6.2 in a positron-emission tomography (PET)-CT (Fig. 1b). Endoscopic ultrasonography-guided fine needle aspiration from the pancreatic tumor was performed and diagnosed as a pancreatic adenocarcinoma by pathological examination. There was no distant organ metastasis. However, because of invasion to the supra-mesenteric artery (SMA), the primary tumor was assessed unresectable. Chemoradiotherapy with gemcitabine (1000 mg/m^2^) was introduced to the patient. After the end of 50.8 Gy radiation, chemotherapy with gemcitabine alone was continued for 13 courses.

**Fig. 2:**
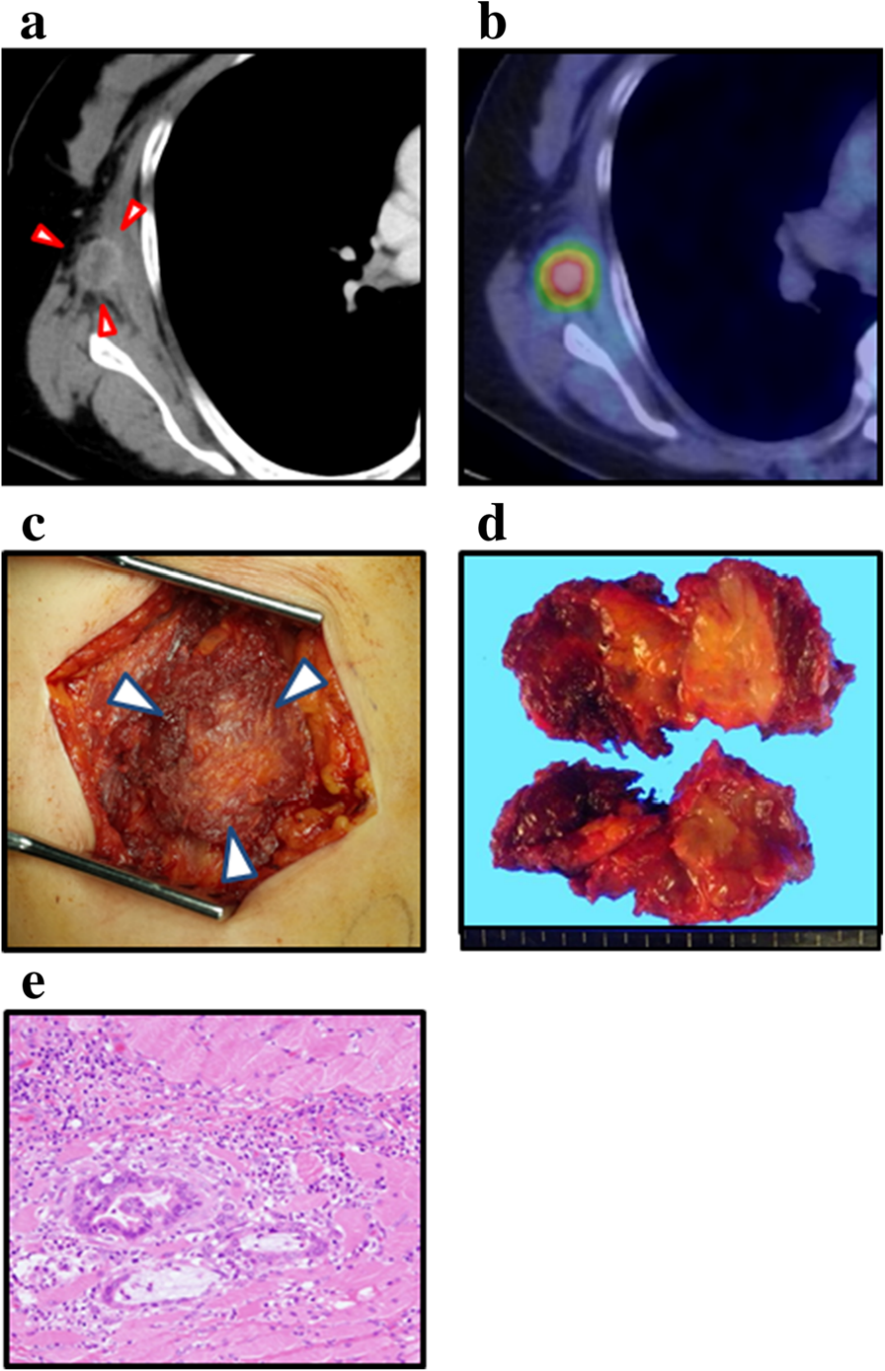
An enhanced CT scan revealed an 18-mm tumor in the right thoracic wall (**a**, arrowheads). PET-CT showed FDG uptake of the tumor (**b**). The tumor existed in the muscle (**c**, arrowheads) and was resected (**d**). Postoperative histological examination confirmed the tumor as a muscle metastasis from the pancreatic cancer (**e**, hematoxylin-eosin staining)

Fifteen months after the induction of the chemoradiotherapy, the size of the primary tumor was reduced from 45 to 25 mm (Fig. 1c). The abnormal uptake of the primary tumor in PET-CT disappeared (Fig. 1d). However, because the primary tumor still involved the SMA, it was not resected. In addition, an 18-mm elastic hard tumor emerged in her right chest wall. An enhanced CT indicated that the tumor existed in the skeletal muscle of the right chest wall (Fig. 2a). The SUV (max) of the tumor was 6.4 in a PET-CT (Fig. 2b). Thus, the chest wall tumor was diagnosed as a metastasis from the pancreatic adenocarcinoma.

There were no other metastatic sites and the primary pancreatic tumor was well controlled. If the chest wall tumor grew, it would cause refractory cancer pain and decrease her quality of life. Avoiding these possible problems, she underwent resection of the tumor of the right thoracic wall. The tumor existed in the trapezius muscle, the teres minor muscle, and the infraspinatus muscle (Fig. 2c). An en bloc resection of the tumor with a part of those muscles was performed (Fig. 2d). There was no postoperative complication. Postoperative histological examination confirmed the tumor as a muscle metastasis from the pancreatic cancer (Fig. 2e).

She had continued chemotherapy with gemcitabine for 6 months after the operation. Then, chemotherapy was changed in S-1 (200 mg/day) and continued 2 months. There was no recurrence at her chest wall. She died due to abdominal dissemination 12 months after the operation.

Metastases into skeletal muscle are rare, with a reported incidence ranging from 0.03 to 0.16% [6–9]. According to literature [6], the most common primary site in SMM is the lung (25.1%), following gastrointestinal tumors (21.0%), but pancreatic cancer is very low which indicated 1% in overall and 5% in gastrointestinal tumors [7]. In addition, some literature showed that SMM occur most commonly in the lower limb, especially in iliopsoas muscle [5]. In English literature, we can found only one case report regarding SMM from pancreatic cancer [10]. Chisthi et al. presented a 55-year-old male patient with complaints of multiple swellings in the right thigh, left shoulder, and both calves [10]. Trucut biopsy from the calf swelling revealed malignant cells amid normal muscle tissue, and the diagnosis was a metastasis from adenocarcinoma. The primary site was searched, and an enhanced CT scan revealed a pancreatic cancer and a liver metastasis. This patient was treated with palliative chemoradiation; however, the authors did not clarify a specific regimen and prognosis of this case [10].

Although blood supply of the skeletal muscles is rich, the reason of the lower frequency of SMM is unclear [11]. Pathophysiological mechanisms of SMM are described in several literature. Most favored pathway is via arterial route [6]. It sounds reasonable that lung cancer is the most common primary site in SMM [5], because lung cancer cells can spread to the skeletal muscles directly by the general circulation system. On the other hand, liver metastasis through portal vein is the most popular distant metastasis in pancreatic cancer [12], and the skeletal muscle is far from the pancreas. Moreover, pancreatic cancer is a highly lethal disease [13], and early metastasis or recurrence such as liver metastasis and dissemination can cause patients’ death before the development of the rare metastases like a SMM. In cancer of pelvic organs, a tumor spreads into the muscle via the paravertebral plexus [14]. The presence of sarcolemma, lactic acid metabolism, tumor suppressors, and lymphocyte may be related to the contractions of muscle tissue [1, 11, 15, 16].

Therapeutic options for SMM may include radiotherapy, chemotherapy, and surgical excision [4]. SMM is generally one of factors for poor prognosis; however, these therapeutic options may help to relieve symptoms, such as pain or swelling. In our case, because of well-controlled general condition and primary tumor, we selected surgical excision, and the patient did not complain about these symptoms until death. On the other hand, it has been not clear whether a removal of a solitary distant metastasis from pancreatic cancer like this case contributes to better prognosis or not.

## Conclusions

The SMM from pancreatic cancer is rare and of poor prognosis. However, in carefully selected patients, we believe that surgical excision may help to relieve symptoms and maintain better quality of life.

## Abbreviations

CT: Computed tomography
PET: Positron-emission tomography
SMM: Skeletal muscle metastasis
SUV: Standardized uptake value
